# Unique Characteristics of the Pyrrolysine System in the 7th Order of Methanogens: Implications for the Evolution of a Genetic Code Expansion Cassette

**DOI:** 10.1155/2014/374146

**Published:** 2014-01-27

**Authors:** Guillaume Borrel, Nadia Gaci, Pierre Peyret, Paul W. O'Toole, Simonetta Gribaldo, Jean-François Brugère

**Affiliations:** ^1^1EA-4678 CIDAM, Clermont Université, Université d'Auvergne, Place Henri Dunant, 63001 Clermont-Ferrand, France; ^2^Department of Microbiology and Alimentary Pharmabiotic Centre, University College Cork, Western Road, Cork, Ireland; ^3^Institut Pasteur, Department of Microbiology, Unité de Biologie Moléculaire du Gène chez les Extrêmophiles, 28 rue du Dr. Roux, 75015 Paris, France

## Abstract

Pyrrolysine (Pyl), the 22nd proteogenic amino acid, was restricted until recently to few organisms. Its translational use necessitates the presence of enzymes for synthesizing it from lysine, a dedicated amber stop codon suppressor tRNA, and a specific amino-acyl tRNA synthetase. The three genomes of the recently proposed Thermoplasmata-related 7th order of methanogens contain the complete genetic set for Pyl synthesis and its translational use. Here, we have analyzed the genomic features of the Pyl-coding system in these three genomes with those previously known from *Bacteria* and *Archaea* and analyzed the phylogeny of each component. This shows unique peculiarities, notably an *amber* tRNA^Pyl^ with an imperfect anticodon stem and a shortened tRNA^Pyl^ synthetase. Phylogenetic analysis indicates that a Pyl-coding system was present in the ancestor of the seventh order of methanogens and appears more closely related to Bacteria than to Methanosarcinaceae, suggesting the involvement of lateral gene transfer in the spreading of pyrrolysine between the two prokaryotic domains. We propose that the Pyl-coding system likely emerged once in Archaea, in a hydrogenotrophic and methanol-H_2_-dependent methylotrophic methanogen. The close relationship between methanogenesis and the Pyl system provides a possible example of expansion of a still evolving genetic code, shaped by metabolic requirements.

## 1. Introduction

Protein synthesis relies on 20 canonical amino acids encoded accordingly to a genetic code, each codon being recognized by an aminoacyl-tRNA. The molecular basis of the genotype to phenotype correspondence relies on the conjunction of tRNAs and of aminoacyl-tRNA synthetases (aaRS). Posttranslational modifications of amino acids extend the chemical nature of proteins with functional implications in cellular processes [1, 2]. Another naturally occurring mechanism expands the genetic code to 22 amino acids by adding Selenocysteine (Sec, 2-selenoalanine) [3, 4] and Pyrrolysine (Pyl, 4-methyl-pyrroline-5-carboxylate linked to the ^*ε*^N of lysine through an amide linkage) [5, 6]. Sec is present in many organisms from the three domains of life [7] with potentially almost a quarter of sequenced bacteria synthesizing it [8]. It is at least present in two orders of Euryarchaeota, the methanogens Methanococcales and Methanopyrales [9, 10]. Its synthesis and incorporation differ from other amino acids, as it is synthesized after a serine has been branched into tRNA^Sec^_UCA_ (recognizing the *opal* codon UGA) that is next modified into a Sec-tRNA^Sec^_UCA_.

In contrast, Pyl is restricted to a very small number of organisms and proteins. It necessitates a complex system with specialized enzymes for biosynthesis of Pyl, a dedicated tRNA and an associated unique aaRS [11, 12]: Pyl is first synthesized as a free amino acid in the cell by the products of the *pylBCD* genes [13, 14] from two lysines, one being methylated into 3-methylornithine (catalyzed by PylB, a lysine mutase-proline-2 methylase) and condensed to the second lysine to form 3-methylornithinyl-N^6^-lysine (PylC, Pyrrolysine synthetase) [15]. The pyrrole ring is then formed by oxidation with an atypical dehydrogenase, the Proline reductase (also called Pyl synthase, PylD) [16], with concomitant release of an amino group during the cycle formation. The gene product of *pylT *forms a dedicated tRNA used for the translational incorporation of Pyl. It is an amber decoding tRNA_CUA_ [6], showing some unusual features compared to other tRNAs [11, 17–19]: the anticodon stem is longer (6 nucleotides instead of 5), while other parts are shorter: D-loop with only 5 bases, acceptor, and D-stems separated by one base instead of 2 and a variable loop of only 3 bases. Moreover, differing from almost all tRNAs, the D-loop does not contain the G_18_G_19_ bases nor the usual T_54_Ψ_55_C_56_ bases in the T-loop. Finally, the aaRS encoded by *pylS* catalyzes the ligation of Pyl to its cognate tRNA species warranting the right correspondence between DNA and proteins. The anticodon of the tRNA seems not to be recognized by the PylRS, at least in *D. hafniense* [20]. Whereas the archaeal PylRS is encoded by a single gene (*pylS*), the bacterial PylRS is encoded by two genes, *pylSn* for the first N-terminal 140 amino acids and *pylSc *for the remaining sequence. This pyrrolysyl-tRNA synthetase belongs to the class II aaRS and its structure has been resolved, with and without its substrates/analogs [17, 21–24]. It has likely a homodimeric or homotetrameric quaternary structure.

To date, Pyl-containing proteins have been found only in the methanogens of the family Methanosarcinaceae and in a few bacteria belonging to Firmicutes and two members of deltaproteobacteria, *Bilophila wadsworthia *and an endosymbiont of the worm *Olavius algarvensis* [25]. Pyl is present almost specifically in the methyltransferases (MT) involved in the methanogenesis pathway from monomethyl-, dimethyl-, and trimethylamine in methanogens, respectively, encoded by the genes *mtmB*, *mtbB*, and *mttB* [26, 27] and in their bacterial homologs, whose function is unclear. However, many bacteria harbor *mttB* homologs that lack the in-frame *amber* codon and the Methanosarcinales *Methanococcoides burtonii *has *mttB* genes with and without an in-frame *amber *codon [11, 25]. In contrast, the *mtbB* gene is almost exclusively present in Methanosarcinales and harbors an in-frame *amber *codon. The *mtmB *gene represents an intermediary case, with a few Pyl-containing bacteria possessing it, leading either to a MT with Pyl (*Acetohalobium arabaticum*, *Desulfotomaculum acetoxidans*) or not (*Bilophila wadsworthia*) [25].

The existence of a 7th order of methanogens related to Thermoplasmatales was proposed from molecular data of 16S rRNA and *mcrA* (methanogenesis marker) sequences retrieved from human stools [28, 29]. This is strengthened by growing molecular data from various environments [30, 31] and by phylogenomics studies [32] established from the first genomes of members of this clade [33, 34]. Moreover, a first member of this order, *Methanomassiliicoccus luminyensis*, has been isolated and grows on methanol + H_2_ [35]. We identified the presence of genes for methanogenesis from methanol and also from dimethylsulfide and methylamines in the two other sequenced genomes (“*Candidatus* Methanomethylophilus alvus” [33], “*Candidatus* Methanomassiliicoccus intestinalis” [36]), together with the Pyl-coding genes and in-frame *amber* codon in the *mtmB*, *mtbB*, and *mttB* genes of these three species. It has now been shown that the H_2_-dependent methanogenesis from trimethylamine is effective for *M. luminyensis* [37]. Also, ruminal methanogens of the same lineage as “*Ca*. M. alvus” (Rumen Cluster C, or RCC), when cultured in consortia with trimethylamine (TMA), show an increase of methanogenesis and mRNAs for MTs, with an *amber* in-frame codon, are detected [38]. Altogether, this greatly suggests the presence of Pyl in these MTs.

The recent uncovered lineage with all the features needed for Pyl encoding and use, representing a new archaeal methanogenic Order, provides an opportunity to better understand the origin, distribution, and diversity of Pyrrolysine-coding systems.

## 2. Materials and Methods

Genomic sequence data were obtained through GenBank. For the 7th order, Thermoplasmata-related methanogens, the accession numbers are CP004049.1 (“*Ca*. Methanomethylophilus alvus”), CP005934.1 (“*Ca*. Methanomassiliicoccus intestinalis”), and NZ_CAJE0100001 to NZ_CAJE0100026 (*Methanomassiliicoccus luminyensis*). For the genomic organization and comparison of *pyl *genes, genomic sequences were either treated with the RAST annotation server [39] or a local Artemis platform [40]. Blast searches [41] were either performed directly on the RAST server, on the nr database at NCBI or locally. Sequence alignment (DNA, RNA, and proteins) was performed using ClustalW [42], T-coffee [43], and MUSCLE [44]. For RNAs, the dedicated R-Coffee program was also used [45], accessible at http://www.tcoffee.org/, which generates a multiple sequence alignment using structural information. RNAfold [46] (available at http://rna.tbi.univie.ac.at/cgi-bin/RNAfold.cgi) and RNAstructure [47] (available at http://rna.urmc.rochester.edu/) were used to determine the secondary structure of tRNA. The structure of the tRNA^Pyl^ was also manually drawn by comparison with the structure of the *D. hafniense* and *M. acetivorans*  tRNA^Pyl^ [11, 20]. Phylogenies were both inferred from maximum likelihood and Bayesian procedures. Datasets of protein homologues were aligned by MUSCLE [44] with default parameters, and unambiguously aligned positions were automatically selected by using the BMGE software for multiple-alignment trimming [48] with a BLOSUM30 substitution matrix. Maximum likelihood trees were calculated by PhyML [49] and the LG amino acid substitution model [50] with 4 rate categories as suggested by the AIC criterion implemented in Treefinder [51]. Trees were also calculated by Bayesian analysis with PhyloBayes [52], with the LG model (for single gene trees) or the CAT model (for the concatenated dataset) and 4 categories of evolutionary rates. In this case, two MCMC chains were run in parallel until convergence and the consensus tree was calculated by removing the first 25% of trees as burnin.

## 3. Results

### 3.1. Genomic Organization of the *pyl* Genes

**Figure 1:**
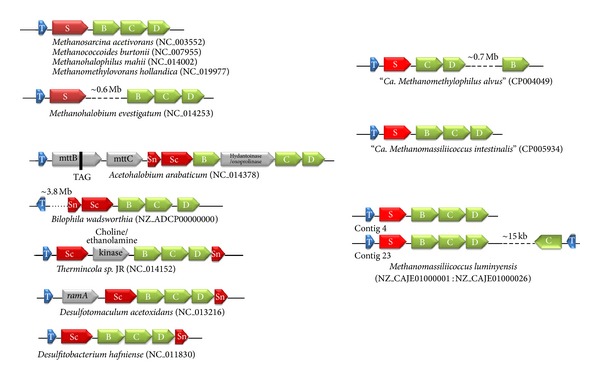
Gene organization of the Pyl system. On the left, the gene organization of the *pylBCD* and  *pylS* (*pylSc *and* pylSn *in bacteria) is shown for Methanosarcinaceae and some representative bacteria (adapted and updated from [11, 13, 53]). On the right is shown the organization of these genes in the 7th order of methanogens.

The *pyl* genes usually occur in close association in archaeal and bacterial genomes [11, 53]. In archaeal genomes, they form a cluster *pylTSBCD* that is not interrupted by other genes, except for *Methanohalobium evestigatum* (Figure 1). In bacterial genomes, the *pyl* genes are generally organized as *pylTScBCDSn* or *pylSnScBCD. *Moreover, the cluster can be interrupted by one or several genes. Each of the three genomes affiliated to 7th order of methanogen displays a distinct organization of the *pyl* genes. “*Ca.* M. intestinalis” has a single *pyl* cluster akin to the general organization observed in most of the Methanosarcinales. Two copies of an identically organized cluster are also found in *M. luminyensis* (contigs 4 and 23), together with a third isolated copy of *pylC* and *pylT* present 15 kb away on the complementary strand (contig 23). In the “Ca. M. alvus” genome, the pyl genes occur in single copy and the pylB gene is ~0.7 Mb distant from the pylTSCD cluster (Figure 1).

Usually, these genes are closely associated with the methylamines MT genes (*mtmB*, *mtbB*, and *mttB*) and other genes involved in these pathways (which do not encode Pyl-containing proteins). A similar gathering of the *pyl* genes cluster with the genes involved in methylotrophic methanogenesis, including *mtmB*, *mtbB*, and *mttB* is observed in the genomes of the 7th order of methanogens (data not shown).

### 3.2. tRNA^Pyl^

**Figure 2:**
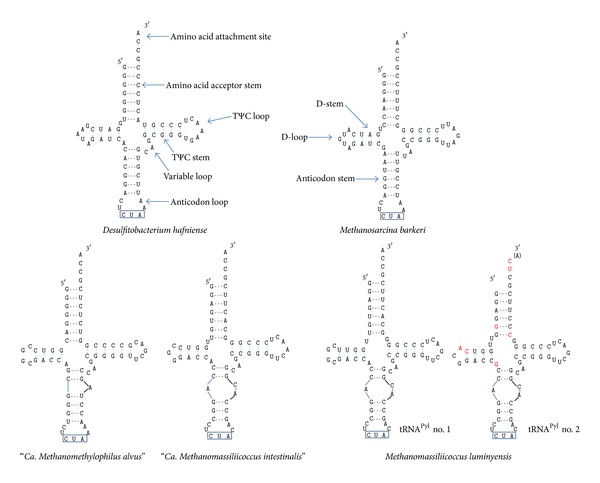
Secondary structure of the tRNA^Pyl^ in the Thermoplasmata-related 7th order. The stem-loop structure of the tRNA^Pyl^ in the 7th methanogenic order is shown, in comparison with the structure in bacteria (*Desulfitobacterium hafniense*, left) and in Methanosarcinaceae (*Methanosarcina barkeri*, right). The name of each region of the tRNA is indicated. The anticodon CUA (corresponding to the *amber *codon) is outlined in blue. For the two tRNA^Pyl^ in *M. luminyensis*, the modified bases in no. 2 compared to no. 1 are written in red. Adapted and updated from [11, 13].

The tRNA^Pyl^ homologues were retrieved from the three 7th order genomes by using the tRNA^Pyl^ of *Methanosarcina barkeri* strain MS-DSM 800 (Accession number AY064401.1) as seed. As mentioned above the “*Ca*. M. alvus” and "*Ca*. M. intestinalis" genomes harbor one *pylT* gene, while three are present in the *M. luminyensis* genome (Figure 1). The third tRNA^Pyl^ of *M. luminyensis* shows no typical stem-loop structures of tRNAs using dedicated bioinformatics tools [54] and is therefore likely a pseudogene. On the contrary, the remaining tRNAs from the three genomes all have a similar shape, different from previously known tRNA^Pyl^ and with stabilities comprised between −28.5 and −23.5 kJ·mol^−1^ (Figure 2). The D-loop already shortened to 5 bases in other tRNA^Pyl^ is here even shorter, with 4 bases in “*Ca*. M. alvus” and in one of the *M. luminyensis* sequences (contig 4, tRNA^Pyl^ no. 1) and even with 3 bases in “*Ca*. M. intestinalis” and in one of the *M. luminyensis* sequences (contig 23, tRNA^Pyl^ no. 2). The acceptor- and D-stems are either not separated (“*Ca*. M. alvus”) or separated by one (“*Ca*. M. intestinalis”; tRNA^Pyl^ no. 2 of *M. luminyensis*) or two bases (tRNA^Pyl^ no. 2 of *M. luminyensis*). The variable loop is conserved in all sequences and is equivalent to that of *D. hafniense*, formed of the three bases CAG. In the anticodon loop, the adjacent base of the anticodon CUA is A in “*Ca*. M. alvus” as observed in *D. hafniense *and *M. barkeri* and C in the two other species. However, the most striking feature is the anticodon stem which is broken in all the tRNA^Pyl^ observed in the 7th order of methanogens. This forms a small loop with a different shape in “*Ca*. M. alvus” and in “*Ca*. M. intestinalis”/*M. luminyensis* (Figure 2).

### 3.3. pyl-tRNA Synthetase

The *pylS* gene encoding the Pyl-tRNA synthetase PylRS is a class II-aaRS (subclass IIc) [21]. The four homologues present in the three genomes are shortened in their 5′ end compared to their Methanosarcinales counterparts (usually around 420–460 AA) and encode, respectively, 275 amino acids in “*Ca*. M. alvus”, “*Ca*. M. intestinalis,” and for one of the two PylRS of *M. luminyensis*, and 271 amino acids (or possibly 308 due to uncertainty of the start codon) for the second. This should lead to an N-ter truncated protein with ~140 residues less, similar to the PylSc proteins that are present in bacteria. We could not identify in any of the three complete genomes a gene encoding a protein similar to these ~140 N-terminal residues present in the Methanosarcinaceae PylS or the bacterial PylSn. Moreover, we found no homologue in these genomes of the particular domain TIGR03912 that is present in archaeal PylS and bacterial PylSn proteins and that enhances the interaction of the tRNA synthetase to its specific tRNA [55]. These particular features of the PylRS may be linked to the peculiar characteristics of the tRNA^Pyl^ and suggest a different kind of interaction.

### 3.4. Phylogenetic Analysis

**Figure 3:**
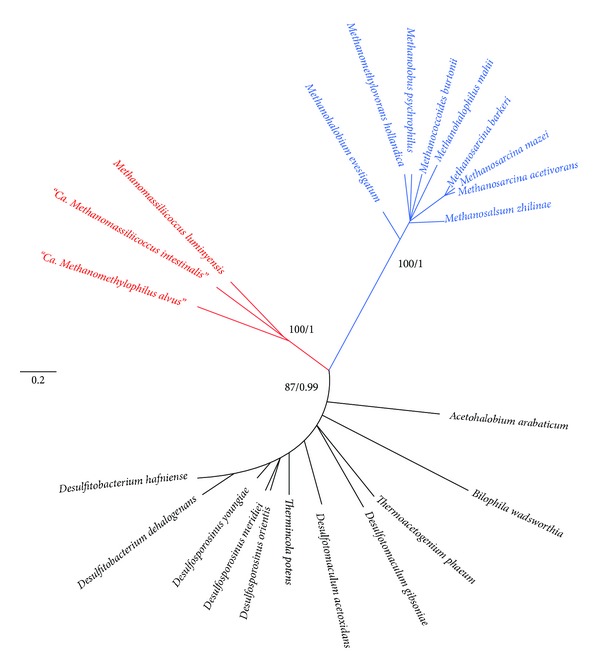
Phylogenetic trees of the *pylSCD *geneproducts. The phylogenetical analysis (PhyML) was performed from a concatenation (672 positions). See Sections 2 and 3 for more details. Individual trees are also reported in the additional file (supplementary Figures S1 to S3 with indication of the corresponding accession numbers). The 7th methanogenic order members are indicated in red, the Methanosarcinaceae in blue, and the bacteria in black.

Phylogenetic analysis was carried out with each of the Pyl gene products by recovering all homologues available in current sequence databases. It appears that bacterial members belong to related Firmicutes species, and a single deltaproteobacterium, *Bilophila wadsworthia*, a common resident of the gut. For PylC, PylD, and PylS, no evident closely related homologues could be found outside of the known Pyl-containing organisms, whereas PylB had a few distant homologues in various bacteria (not shown). Pyl proteins are very well conserved at the sequence level among *Archaea* and *Bacteria* (alignments are available on request from the authors). The PylC, PylD, and PylS datasets gave consistent results (additional files, Figures S1 to S3 available online at http://dx.doi.org/10.1155/2014/374146) and were therefore concatenated to provide increased phylogenetic signal (Figure 3). “Ca. M. alvus”, “Ca. M. intestinalis,” and “*M. luminyensis*” form a robustly supported monophyletic cluster, indicating a common origin of the Pyl system in the 7th methanogenic order. The products of the two *pyl* gene clusters of *M. luminyensis* (including the third extra PylC) are more closely related among them than to the other sequences, indicating that they originated from a specific duplication. Methanosarcinaceae and bacterial sequences also form two distinct and robustly supported monophyletic clusters. Moreover, the sequences from the 7th methanogenic order appear to be more closely related to their bacterial counterparts than to Methanosarcinaceae (Figure 3).

Concerning PylB, phylogenetic analyses gave more ambiguous results, with a poorly supported branching of the 7th methanogenic order within Bacteria, and different inferred evolutionary relationships according to exclusion or inclusion of the distant bacterial outgroup (data not shown). An unrooted PhyML phylogenetic tree inferred exclusively from PylB (without the distant bacterial outgroup) is given in the additional file Figure S4.

## 4. Discussion

We have shown here that all the genetic components for Pyl synthesis and use are present in a new order of Archaea performing methylotrophic methanogenesis from methanol and methylamines, therefore enlarging the taxonomic distribution of this genetic code expansion cassette. Moreover, this Pyl system has unique features and appears more closely related to the bacterial one than to those present in Methanosarcinaceae. Whether or not the Pyl system is active in these archaeons is beyond the scope of this paper. However, there are many arguments supporting a functional system. These include, for the three genomes available and analyzed to date, at least the global presence of all the genes for Pyl synthesis, charging and cotranslational incorporation, in cooccurrence with all the genes for methylotrophic methanogenesis from methylamines, that is, those coding for methylamines-corrinoid protein methyltransferases (MT) genes (*mtmB, mtbB*, and* mttB*) all bearing an in-frame *amber* codon. It is interesting to note that, as a nonsense (stop) codon, the *amber* codon is used with a low frequency, this value being maximal in the *M. luminyensis* genome, with only 11% of stop codon being an amber one. It is likely an adaptive strategy to the presence of the Pyl system in this order, similarly to what is observed in Methanosarcinaceae, but different from Bacteria: these lasts deal with the presence of Pyl through regulation rather than codon avoidance [25]. Ability to metabolize TMA into methane has been shown for *M. luminyensis* [37]. Moreover, it has also been shown that methylotrophic methanogenesis from methylamines is active in the rumen, very likely carried out by Rumen Cluster C, members of the 7th order of methanogens which are close neighbors or similar to “*Ca*. M. alvus” [38]. This is therefore conceivable that methylotrophic methanogenesis is constitutive of this whole order by dedicated MTs containing Pyl. The presence of unique features in the Pyl system in these archaeons raises the exciting question of how it functions. PylRS harbors a shorter form, more closely related to the product of the bacterial pylSc gene than to the C-terminus of the PylS present in Methanosarcinaceae, and lacks the ~140 amino acids equivalent to the bacterial *pylSn *or to the N-terminal of the archaeal PylS. Bacterial PylSn and the Nter part of Methanosarcinaceae PylRS contain the protein domain TIGR03912 [55]. In the three genomes of the 7th order of methanogens, no CDS containing this peculiar domain was found by an InterProScan analysis [56]. The lack of these elements in the tRNA^Pyl^ synthetase (PylSn-TIGR03912 domain) indicate that PylRS must solely rely on a homomeric structure and might be able to fulfill this role without the domain corresponding to PylSn. Indeed, it has been shown that the *D. hafniense* PylSc is alone sufficient to charge a cognate tRNA^Pyl^ with an analogue of pyrrolysine in a recombinant system [20]. Alternatively, it may rely on the presence of a yet unknown enhancing component. Also exciting are the sequence and inferred structure of the tRNA^Pyl^: its extremely condensed nature, with a peculiar broken anticodon stem is of concern for future functional and evolutionary studies, together with the molecular mechanisms which sustain this orthogonal code between the tRNA^Pyl^ and the PylRS. The peculiar structure of the tRNA^Pyl^ may strengthen the interaction with its cognate PylRS and be an adaptation following the loss of the PylSn domain. Alternatively, mutations in the sequence of the tRNA^Pyl^ may have led to the loss of the PylSn. However, it is also possible that this domain was never present in the Pyl system of these archaeons. In any case, active or not, the well-supported monophyly of the Pyl encoding genes in the three genomes argue for their presence in their common ancestor. Because these represent at least two distinct clades [32], this indicates the likely presence of a Pyl system early in the 7th order of methanogens or even in the ancestor of the whole order. The same conclusion stands also for the Methanosarcinaceae.

How the 22nd proteogenic amino acid Pyrrolysine was added to the genetic code is an exciting question. Based on experimental data, it has been proposed that the universal codon catalog was anterior to the aminoacylation systems [57]. 3D structure-based phylogeny of PylRS has led to the suggestion that it was already present in the last universal common ancestor (LUCA) of all present-day life beings and has a common origin with the phenylalanine tRNA synthetase PheRS [21]. If true, this would mean that Pyl was inherited from the LUCA and retained only in the few present-day methylamines-utilizing bacterial and archaeal species. Similarly, Fournier and colleagues proposed that Pyl originated in a pre-LUCA lineage representing a yet unidentified or more probably extinct 4th domain of life [58–60]. In this hypothesis, Pyl would have been acquired in a few *Bacteria* and *Archaea *via several independent lateral gene transfers (LGT) from members of this hypothetical 4th domain of life. However, its highly specific function and restricted distribution make it more likely that the addition of Pyl is as a more recent event.

The strong link of the Pyl system with methanogenesis, especially the methylotrophic one, argues for a common evolutionary history. Pyl is now essential for methylotrophic methanogenesis from methylamines, while methylotrophic methanogenesis from methanol is independent of Pyl. Pyl has been found in *Archaea *almost exclusively in the methyltransferases involved in methanogenesis from mono-, di-, and tri-methylamines (MtmB, MtbB, and MttB). Moreover, *pyl *genes and *mtm-mtb-mtt *genes are clustered in the genomes of methylamines-utilizing archaeal* species*; therefore, a stimulating hypothesis is that genes necessary for Pyl synthesis/incorporation and for methylotrophic methanogenesis from methylamines have coevolved, whereas this was not the case for methylotrophic methanogenesis from methanol, which is independent of pyrrolysine-containing enzymes.

Methanogenesis is believed to have arisen early in the evolution of Euryarchaeota, likely after the divergence of Thermococcales, and to have been lost several times independently in various lineages (e.g., Halobacteriales, Archaeoglobales, and Thermoplasmatales) [32, 61, 62]. The first form of methanogenesis might have been the hydrogenotrophic one (present in the class I methanogens composed of Methanopyrales, Methanobacteriales, and Methanococcales), while other types of methanogenesis (methylotrophic non-H_2_-dependent and acetoclastic, involving cytochromes) would have emerged later. Based on phylogenomic studies, we have recently shown that the pathway for methylotrophic methanogenesis, at least from methanol, was already present in the ancestor of Methanobacteriales, in the ancestor of the 7th order, and in the ancestor of Methanosarcinales, which is the common ancestor of most euryarchaeal lineages excluding Thermococcales [32]. Therefore, H_2_-dependent methylotrophic methanogenesis from methanol may also be an ancient type of methanogenesis. The fact that it does not involve cytochromes and needs fewer genes could favor such hypothesis. The question is, can the same conclusions be made for the methylotrophic methanogenesis from methylamines? The fact that this methanogenesis is presently restricted to Methanosarcinaceae and the 7th order of methanogens, which emerged after class I methanogens, argues for a more recent event. It has also to be stressed that no *mta* gene (involved in methylotrophic methanogenesis from methanol) harbors an *amber*-Pyl codon. Moreover, the last MT enzyme of the methanogenesis pathway (encoded by *mtaA *gene for methanol) that precedes the steps corresponding to core methanogenesis (methyl-coM reduction to generate methane) is involved in methanogenesis from both methanol and some methylamines in Methanosarcinaceae and likely also in the 7th order (data not shown). Therefore, it is tempting to speculate that the Pyl-independent, H_2_-dependent methanogenesis from methanol preceded the Pyl-dependent, H_2_-dependent methylotrophic methanogenesis from methylamines.

**Figure 4:**
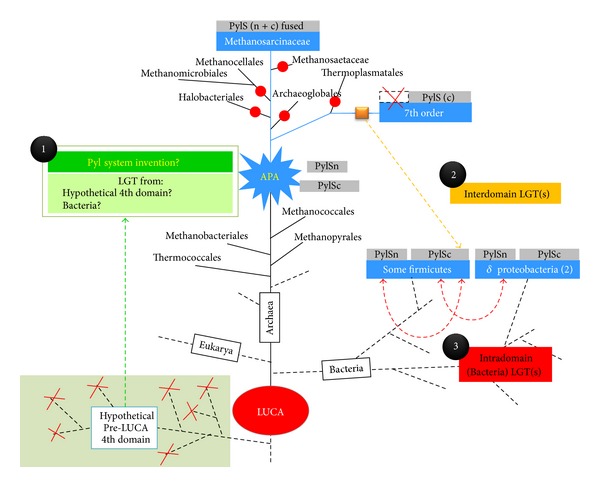
Proposed scenarios for the origin and evolution of the pyrrolysine system. One of the various possible models leading to the genesis of APA, the ancestral Pyl-coding archaeon, is schematized. This model supports less LGTs than previously proposed ones. APA (blue star) emerged in the Euryarchaeota (black circle no. 1) after the birth of the methanogenesis and was an ancestor of the Methanosarcinalesand/or of the Thermoplasmata-related 7th order of methanogens. Only the hypothesis of a common ancestor of both Methanosarcinalesand Thermoplasmata-related 7th order of methanogens is depicted here (see text for an alternative model with APA as a more recent ancestor, of the Methanosarcinalesor of the Thermoplasmata-related 7th order of methanogens, but not both). The putative genesis of APA (green boxes) relies either from an archaeal invention (Box 1), from a bacterial contribution, or from a whole functional system acquired from a hypothetical pre-LUCA 4th domain of life. APA was therefore likely a methanogenic archaeon performing both a methanol-H_2_-dependent methylotrophic methanogenesis and a hydrogenotrophic one. The Pyl system was vertically inherited (symbolized by large blue lines) to Methanosarcinaceae and to the 7th methanogenic order and was lost several times independently on various branches, including methanogenic ones (red dots). Due to the closest relationship of the Pyl system (orange square) between the 7th order and bacteria, at least one LGT is supposed to have occurred from a 7th order ancestor (before the loss of the *pylSc *gene in this lineage), likely to a Firmicute(no. 2, orange arrow). Other LGTs from the 7th order to Bacteria (i.e., a deltaproteobacterium) are also conceivable. In a third period, intradomain LGT(s) could have originated next putatively among Firmicute and/or into a deltaproteobacterium (no. 3, red arrows). Pyl-coding organisms are symbolized in blue boxes. The nature of the PylRS is depicted with each Pyl-containing group in grey boxes and is either formed by a unique PylS (Methanosarcinaceae), a split complete version (PylSc//PylSn in bacteria), or a unique PylSc form (7th methanogenic order).

Taking together these pieces of evidence, it is likely that, in the archaeal domain, the Pyl system arose in a methanogenic euryarchaeote, in correspondence to the emergence of the genes coding for methylamines-corrinoid protein methyltransferases (MT) (*mtmB*, *mtbB*, and* mttB*) that all bear an in-frame *amber* codon, allowing the use of new substrates (mono-, di-, and trimethylamine, resp.). We will refer to the archaeon where the system arose as the archaeal Pyrrolysine ancestor (APA). The APA may correspond to the ancestor of Methanosarcinaceae, the ancestor of the 7th order of methanogens, or the common ancestor of both (depicted on Figure 4). Now, it can be asked how the Pyl system arose in this hypothetical euryarchaeote. Two possibilities can be envisaged: (i) it was acquired *via* a lateral gene transfer (LGT) or (ii) it emerged autogenously.

Concerning the hypothesis of an LGT from a 4th domain of life by Fournier and colleagues [58, 59], in the light of our results it may be reformulated by positing a single LGT from this extinct lineage into Archaea after the divergence of Thermococcales to give birth to the APA (Figure 4, dotted green arrow). The system would have then been retained only in present-day pyl-containing organisms, for example, the 7th order and Methanosarcinaceae, whereas it would have been lost multiple times independently in most euryarchaeal lineages, including methanogenic ones (Figure 4, red dots). However, the hypothetical existence of a pre-LUCA lineage that gave rise to the Pyl system remains questionable because of the necessary coexistence of the donor and the APA and because the LGT would have taken place in a euryarchaeon, relatively recent and distant from LUCA, the 4th domain would have been composed at that time of many different lineages that would have subsequently all disappeared (broken dotted lines in the hypothetical 4th Domain, Figure 4).

Our data also make the hypothesis that the Pyl system arose in Bacteria and was introduced in the APA via LGT less likely (Figure 4). In fact, its presence in the 7th order now makes its taxonomic distribution much larger in Archaeathan the few pyl-containing bacteria representing a sublineage of Firmicutes and two deltaproteobacteria and its link to methylotrophic methanogenesis stronger.

Therefore, our preferred hypothesis is that the Pyl system is an archaeal invention (green box, Figure 4). PylRS might have emerged by gene duplication followed by fast evolutionary rates from another class II RS gene, while a mutation in the anticodon of a duplicated tRNA could have led to an *amber *decoding tRNA. This would have had less detrimental effects for the cell than the recoding of another codon, affecting potentially all the proteins of the cell. Interestingly, PylRS does not need to recognize the anticodon of the tRNA^Pyl^ [17, 55], and this may be a remnant of the birth of the orthogonal pair tRNA^Pyl^/PylRS. Moreover, the recent discovery of the whole synthesis pathway of Pyl, with PylB being a lysine mutase [13] shows that Pyl is entirely a derivative of a proteogenic amino acid (two lysines) and this could make sense in the light of the coevolution theory, such are the cases of Asp/Asn and Glu/Gln in Archaea[63]. APA may have been an early diverging euryarchaeote performing H_2_-dependent methanogenesis from methanol, perhaps the ancestor of Methanosarcinales and the 7th order of methanogens and therefore after the emergence of Thermococcales and methanogens class I (Methanococcales, Methanobacteriales, and Methanopyrales). Vertical inheritance of the Pyl system would have paralleled that of H_2_-dependent methanogenesis from methylamines whereas multiple independent losses would have occurred over time in most euryarchaeal lineages, including methanogenic ones (Figure 4, red dots), but retained only in the Methanosarcinaceae and the 7th order. Alternatively, the system would have emerged later, in either the ancestor of Methanosarcinaceae or in the ancestor of the 7th order and then spread among them via LGT (not indicated in the Figure 4 for clarity). Because their Pyl systems are different (notably the absence of the N-terminus of PylS and the unique peculiarity of the tRNA^Pyl^), it may be asked which on the two is the ancestral one. However, it is likely that the unique system of the 7th order is derived from a more “classical” one such as those present in bacteria and Methanosarcinaceae.

Under the hypothesis of an archaeal invention of the Pyl system, the bacterial one may have arisen via LGT from the 7th order of methanogens, considering their closer evolutionary relations, before the loss of the N-terminus of PylS (Figure 4, PylS structure indicated above the Pyl-containing groups). It is unclear if one or several LGTs occurred from the 7th order onto bacteria (Firmicutes and deltaproteobacteria). The group of Firmicutes is likely the recipient of this first transfer and would have then given the system to a deltaproteobacterium. Alternatively, due to their close environmental relationship (the gut), a distinct LGT could have arisen from a 7th order member of the gut into *Bilophila sp*. However, it cannot be excluded that bacteria took it from Methanosarcinaceae and split the PylS gene into PylSn and PylSc and then they later passed only PylSc to the 7th order. The tree of PylB may suggest such hypothesis, albeit statistical support is not very strong and no similar pattern is observed for the other components of the system. Unfortunately, phylogenetic analysis prevents concluding the direction of these transfers, because of the absence of outgroup sequences. In every case, that these inter- and/or intradomain LGTs were not rejected is a fascinating event. Introducing a stop codon suppressor is in fact likely deleterious and has to be compensated by a strong selective advantage for the organism, such as the use of new metabolic substrates (e.g., methylamines). Introducing a Pyl-coding cassette into a heterologous genome has been successfully experimentally realized [14, 64]. It is possible that the acquired genes remained silent or with a low expression level and would have been activated progressively, leading concomitantly to the negative selection of UAG nonsense codons. A more likely hypothesis is an LGT leading to an inducible expression of the Pyl components, such as dependence of the presence of substrates like methylamines. The natural expansion of the genetic code in the Firmicute *Acetohalobium arabaticum* able to genetically encode the 20 usual amino acids when grown on pyruvate, and to expand its repertoire to 21 by adding pyrrolysine when grown on TMA [25] provides the paradigm.

In conclusion, when considering at least the archaeal domain, there has been a Pyl-coding ancestor. It appeared likely relatively recently, as an ancestor of the Methanosarcinales, an ancestor of the 7th order of methanogens, or the common ancestor of both. It was likely a methanogen performing a methanol/H_2_-dependent methanogenesis and, considering the probable coevolution history between methylotrophic methanogenesis and Pyl, with their strong interdependence, it was concomitant or rapidly followed by the emergence of methanogenesis from methylamines compounds. This has led nowadays in the archaeal lineage to conservation of a Pyl system only in methylamines-utilizing/Pyl-dependent methanogens. Therefore, this provides an example that the genetic code may be still under evolution with a conceivable expansion shaped by metabolic requirements.

## Supplementary Material

Supplementary Material: displays the individual phylogenies respectively of PylC, PylD, Pyl S and PylB (Figures S1 to S4). Briefly, phylogenies were inferred from maximum likelihood procedures: Datasets of protein homologues (strains and accession numbers on each figure) were aligned by MUSCLE [44] and unambiguously aligned positions were automatically selected by using the BMGE software [48] with a BLOSUM30 substitution matrix. Maximum likelihood trees were calculated by PhyML [49]. See the Materials and Methods section for more details.
