# Corrigendum to “Unique Characteristics of the Pyrrolysine System in the 7th Order of Methanogens: Implications for the Evolution of a Genetic Code Expansion Cassette”

**DOI:** 10.1155/2015/941836

**Published:** 2015-02-02

**Authors:** Guillaume Borrel, Nadia Gaci, Pierre Peyret, Paul W. O'Toole, Simonetta Gribaldo, Jean-François Brugère

**Affiliations:** ^1^EA-4678 CIDAM, Clermont Université, Université d'Auvergne, Place Henri Dunant, 63001 Clermont-Ferrand, France; ^2^Department of Microbiology and Alimentary Pharmabiotic Centre, University College Cork, Western Road, Cork, Ireland; ^3^Institut Pasteur, Department of Microbiology, Unité de Biologie Moléculaire du Gène chez les Extrêmophiles, 28 rue du Dr. Roux, 75015 Paris, France

In the paper titled “Unique Characteristics of the Pyrrolysine System in the 7th Order of Methanogens: Implications for the Evolution of a Genetic Code Expansion Cassette,” a mistake arose in* M. luminyensis *tRNA^Pyl^ sequences shown in the lower part of Figure 2. In this erratum, we report the corrected Figure 2 with the correct tRNA^Pyl^ sequences.

## Figures and Tables

**Figure 2:**
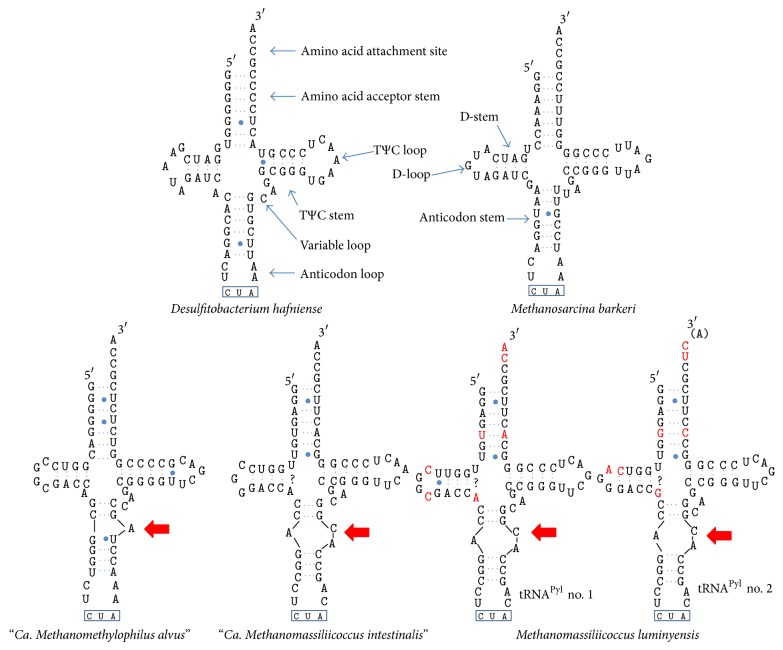
Secondary structure of the tRNA^Pyl^ in the Thermoplasmata-related 7th order of methanogens. The stem-loop structure of the tRNA^Pyl^ in the 7th order of methanogens is shown, deduced by comparison with the structure of this tRNA in bacteria (*Desulfitobacterium hafniense*, top left) and in Methanosarcinaceae (*Methanosarcina barkeri*, top right), adapted from [11, 13]. The name of each region of the tRNA is indicated in the upper part. The anticodon CUA (corresponding to the* amber* codon) is outlined in blue. The red arrows indicate the shortened anticodon stem in the 7th order of methanogens broken by unpaired bases that form an unusual loop in this region. The question marks represent other possible base pairing. For the two tRNA^Pyl^ in* M. luminyensis*, the modified bases between no. 1 and no. 2 are written in red.

