# Supplementary material for: Unique Characteristics of the Pyrrolysine System in the 7th Order of Methanogens: Implications for the Evolution of a Genetic Code Expansion Cassette
Source: Archaea. 2014 Jan 27;2014:374146. doi: 10.1155/2014/374146 (PMC3941956; doi:10.1155/2014/374146)
Supplement: Supplementary file 1 — Supplementary Material: displays the individual phylogenies respectively of PylC, PylD, Pyl S and PylB (Figures S1 to S4). Briefly, phylogenies were inferred from maximum likelihood procedures: Datasets of protein homologues (strains and accession numbers on each figure) were aligned by MUSCLE [44] and unambiguously aligned positions were automatically selected by using the BMGE software [48] with a BLOSUM30 substitution matrix. Maximum likelihood trees were calculated by PhyML [49]. See the Materials and Methods section for more details. [file 374146.f1.pdf]

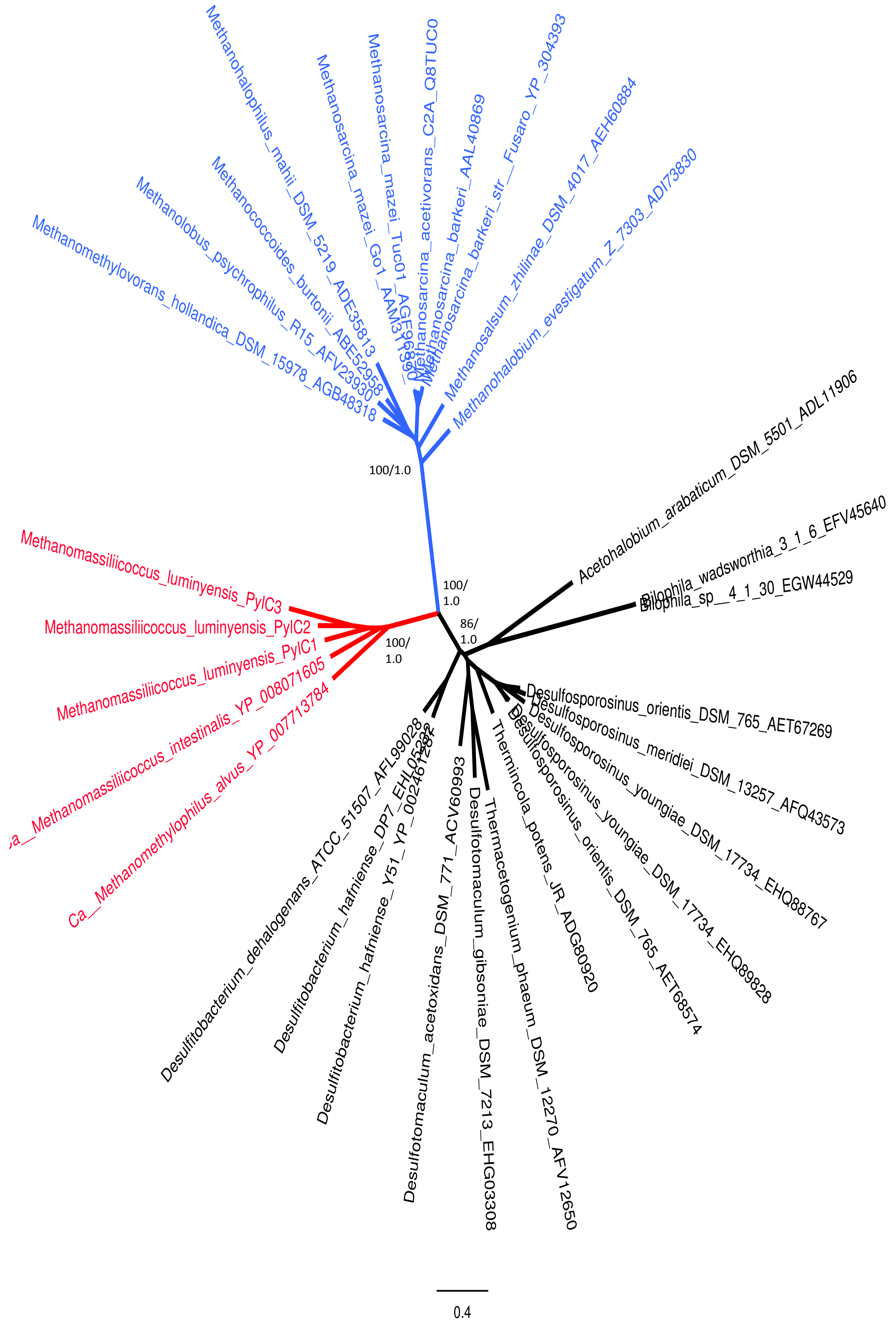

Figure S1: PylC phylogeny (PhyML)

In black, baterial PylC – in blue, Methanosarcinaceae PylC – in red, 7th methanogenic order PylC

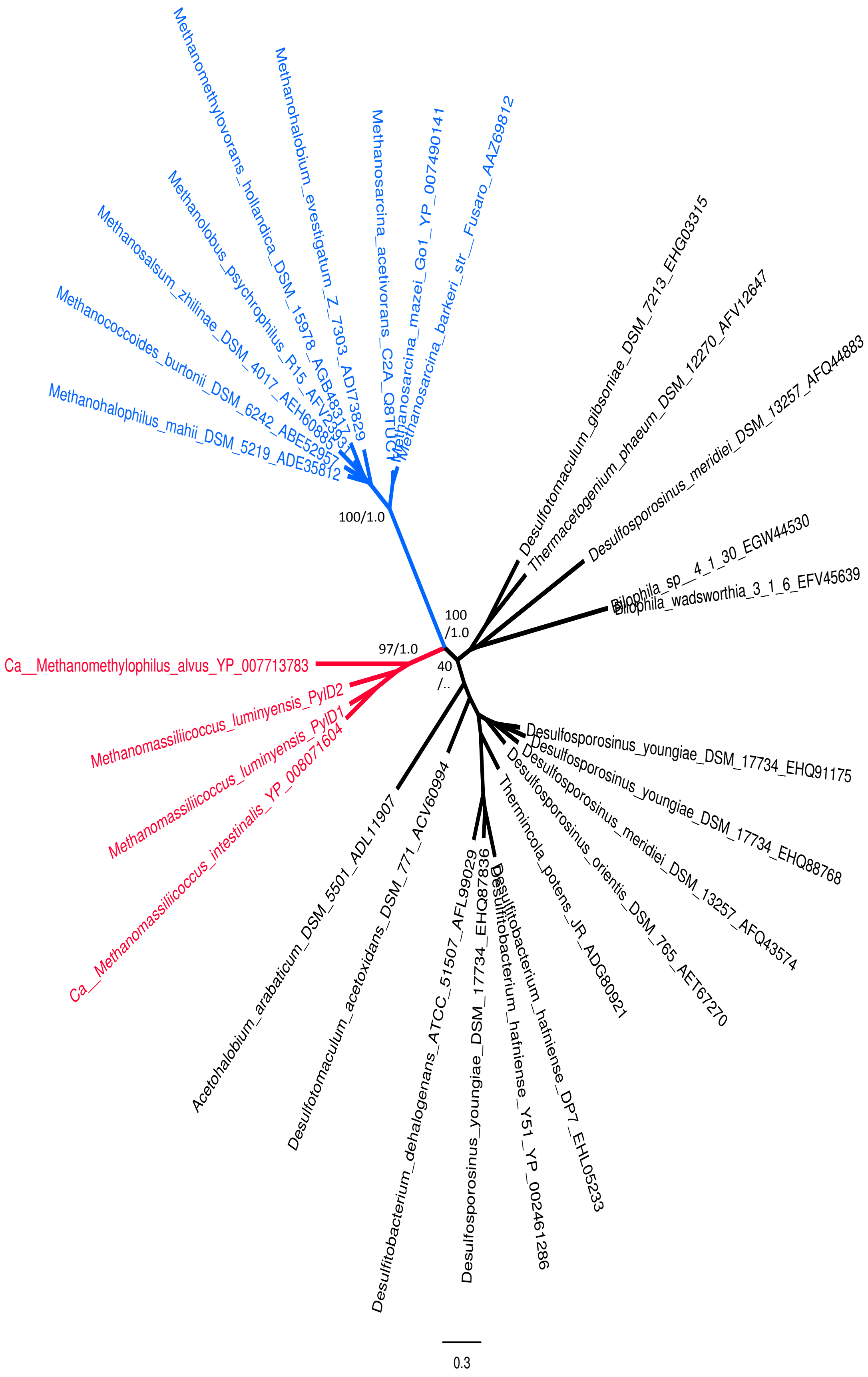

Figure S2: PylD phylogeny (PhyML)

In black, baterial PylD – in blue, Methanosarcinaceae PylD – in red, 7th methanogenic order PylD

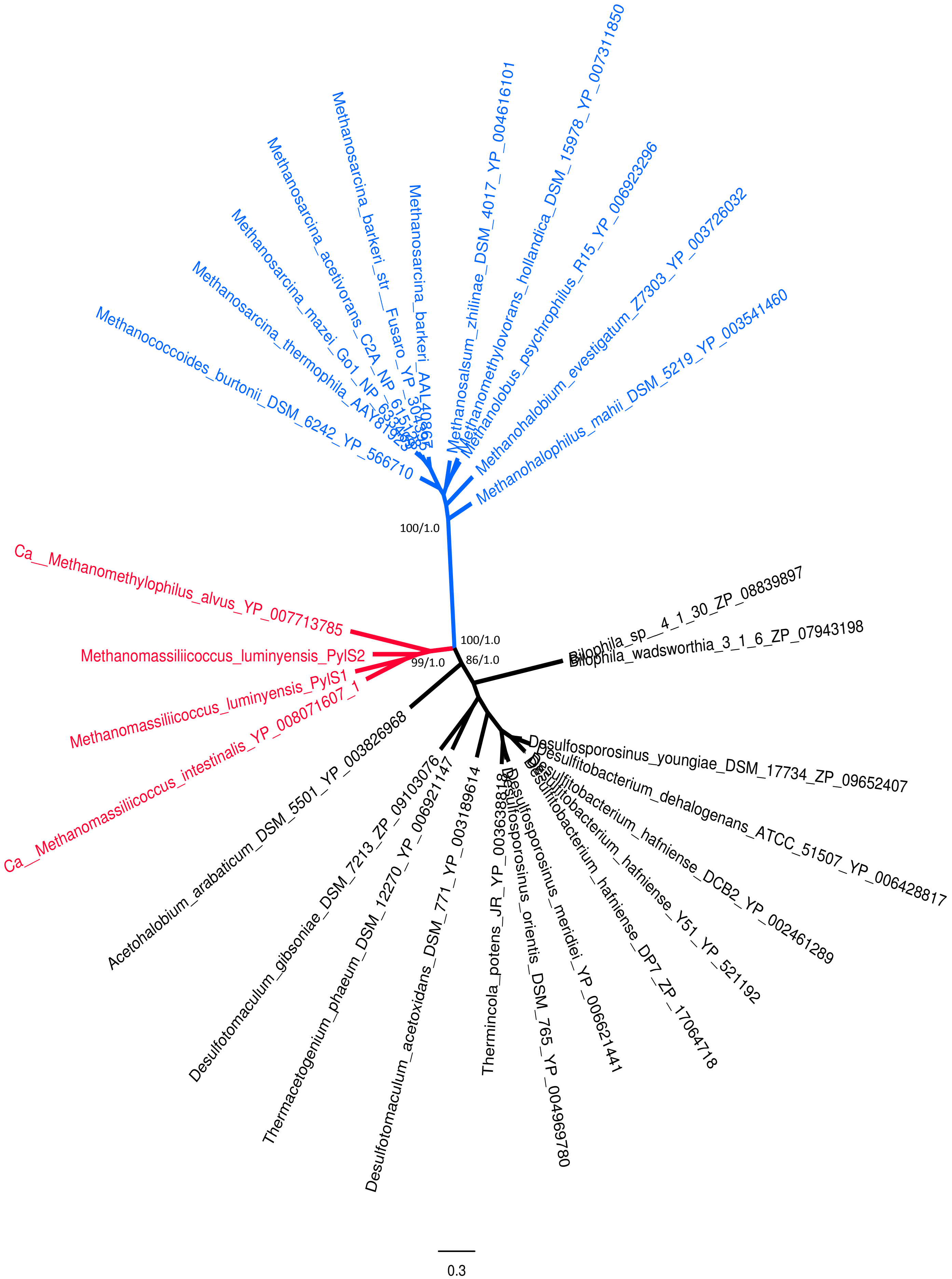

Figure S3: PylS phylogeny (PhyML)

In black, baterial PylS – in blue, Methanosarcinaceae PylS – in red, 7th methanogenic order PylS

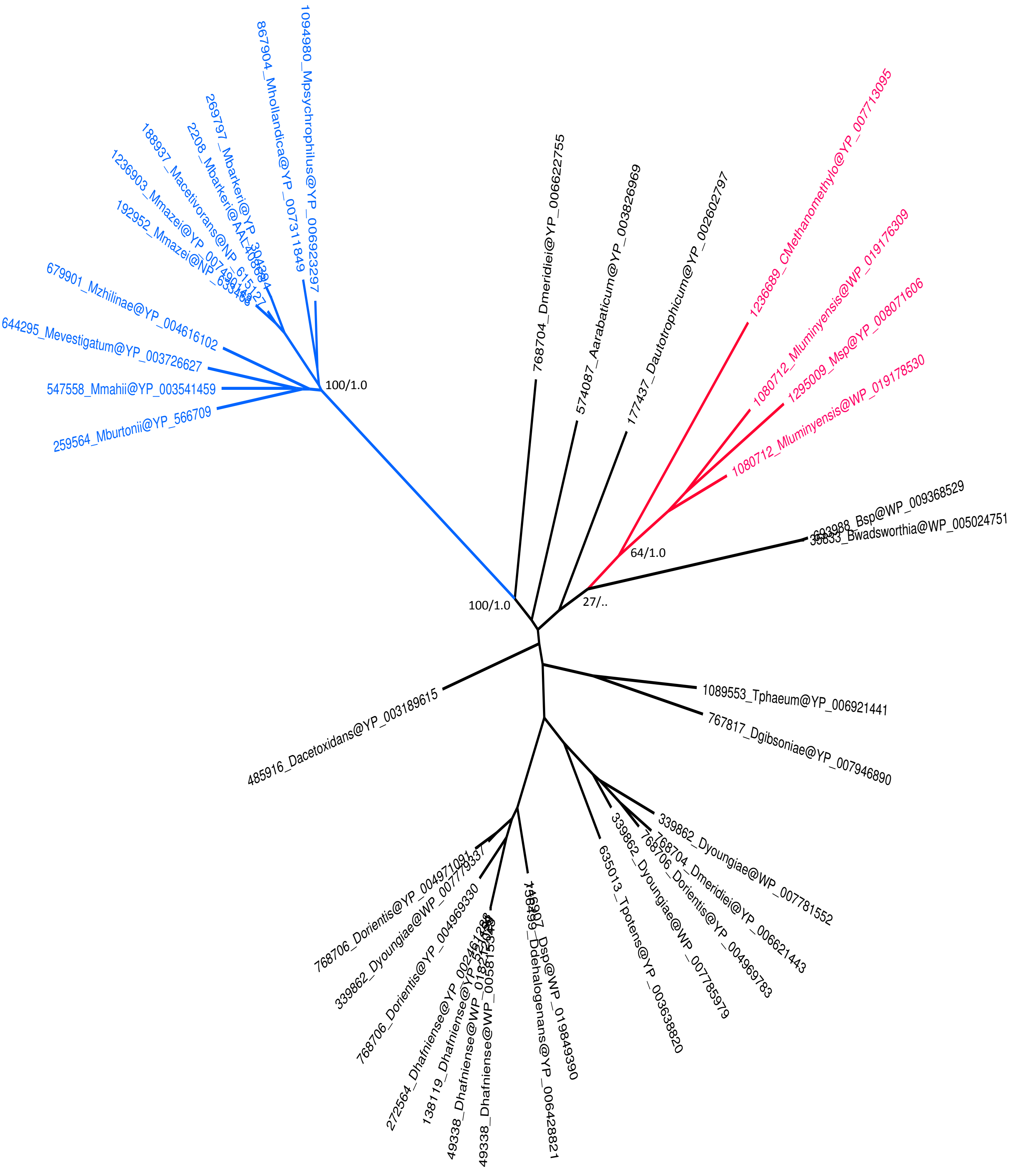

Figure S4: PylB phylogeny (PhyML)

In black, baterial PylB – in blue, Methanosarcinaceae PylB – in red, 7th methanogenic order PylB
